# Impact of Cigarette Smoking on the Gastrointestinal Tract Inflammation: Opposing Effects in Crohn’s Disease and Ulcerative Colitis

**DOI:** 10.3389/fimmu.2018.00074

**Published:** 2018-01-30

**Authors:** Loni Berkowitz, Bárbara M. Schultz, Geraldyne A. Salazar, Catalina Pardo-Roa, Valentina P. Sebastián, Manuel M. Álvarez-Lobos, Susan M. Bueno

**Affiliations:** ^1^Millennium Institute on Immunology and Immunotherapy, Departamento de Genética Molecular y Microbiología, Facultad de Ciencias Biológicas, Pontificia Universidad Católica de Chile, Santiago, Chile; ^2^Departamento de Gastroenterología, Facultad de Medicina, Pontificia Universidad Católica de Chile, Santiago, Chile

**Keywords:** cigarette smoking, gastrointestinal inflammation, inflammatory bowel disease, ulcerative colitis, Crohn’s disease

## Abstract

Cigarette smoking is a major risk factor for gastrointestinal disorders, such as peptic ulcer, Crohn’s disease (CD), and several cancers. The mechanisms proposed to explain the role of smoking in these disorders include mucosal damage, changes in gut irrigation, and impaired mucosal immune response. Paradoxically, cigarette smoking is a protective factor for the development and progression of ulcerative colitis (UC). UC and CD represent the two most important conditions of inflammatory bowel diseases, and share several clinical features. The opposite effects of smoking on these two conditions have been a topic of great interest in the last 30 years, and has not yet been clarified. In this review, we summarize the most important and well-understood effects of smoking in the gastrointestinal tract; and particularly, in intestinal inflammation, discussing available studies that have addressed the causes that would explain the opposite effects of smoking in CD and UC.

## Introduction

Cigarette smoking is a major risk factor for the development of vascular diseases, such as atherosclerosis and pulmonary hypertension (1). Moreover, smoking-related diseases are the leading cause of preventable deaths and respiratory dysfunctions worldwide. Not only respiratory and cardiovascular diseases contribute to the morbidity and mortality associated with smoking but also other conditions such as multiple inflammatory and carcinogenic disorders (2). The underlying cause of these diseases is the presence of a large number of toxic components in cigarette smoke, whose impact at the respiratory and vascular level has been widely studied (1). Nevertheless, the pathways that are affected by cigarette smoke within the gastrointestinal tract, especially in inflammatory bowel disease (IBD), have not been clarified.

This review will focus on the impact of cigarette smoking on the gastrointestinal system, focusing on recent studies that have addressed its opposite effects on the two major forms of IBD: Crohn’s disease (CD) and ulcerative colitis (UC).

### General Harmful Effects of Cigarette Smoking

The main component of cigarette, tobacco, is a natural product with a complex molecular composition. During smoking, this complex biomass is subjected to high temperatures and varying oxygen concentrations, producing an incomplete combustion that generates more than 7,000 toxic compounds. Cigarette smoke can be divided into mainstream and sidestream smoke. Mainstream smoke is the fraction that the smoker inhales directly, and is composed of a particulate phase and a gas phase (3). Low molecular weight components, such as carbon monoxide (CO) and light aldehydes, are the main substances present in the gas phase, which immediately enters the pulmonary circulation (4). On the other hand, compounds such as nicotine, polycyclic aromatic compounds, nitrosamines, and heavy metals are predominantly found in the particulate phase, which is absorbed by mucous membranes, skin, alveoli, and the gastrointestinal system (5).

It has been shown that chronic inhalation of cigarette smoke alters cell proliferation, endothelial function (1), and immune response (6). To date, more than 60 carcinogenic and mutagenic compounds have been identified in cigarette smoke, most of them in the particulate phase. Among the most dangerous compounds are some polycyclic aromatic hydrocarbons and *N*-nitrosamines (7). In addition, cigarette smoke has high levels of reactive oxygen species, peroxynitrite, peroxynitrate, free radicals, and reactive organic compounds that produce oxidative stress and modulate nitric oxide-dependent endothelial function (4). In fact, both smokers and animals exposed to cigarette smoke have markers of systemic oxidative stress (8), which can negatively affect susceptible tissues, such as the gastrointestinal tract.

### Effects of Cigarette Smoke on the Gastrointestinal Tract

In addition to the respiratory and cardiovascular effects produced by cigarette smoke, several studies suggest that smoking is also harmful to the gastrointestinal tract, as summarized below (Table 1). Cigarette smoke contains between 10^14^ and 10^16^ free radicals per puff (9), both in the gas phase and in the particulate phase. The active chemicals include aldehydes, quinones, benzo(a)pyrene, epoxides, and peroxides (3), which can induce the production of reactive oxygen species. If these are not neutralized by antioxidants, oxidative stress occurs, which causes tissue damage. Accordingly, it has been shown that cigarette smoking increases the incidence and relapse of peptic ulcer disease (10), and has been associated with IBD, CD, and the development of esophageal, stomach, liver, pancreatic, and colon cancer (5).

**Table 1:** Effects of cigarette smoke on the gastrointestinal tract.

Effect of cigarette smoke	Associated disease	Reference
**Lumen composition (mucus and microbiota)**		
Inhibition of mucus synthesis by interference with epidermal grow factor (EGF) expression and polyamines synthesis.	Gastric and peptic ulcer	(10, 11)
Alteration on mucus composition, related with an increment on Muc2 and Muc3 expression in the ileum and Muc4 expression in the colon.	IBD	(12)
Dysbiosis of gut resident microbiota, with an increase of Firmicutes and Actinobacteria and a decrease of Bacteroidetes and Proteobacteria.	IBD	(13, 14)
**Integrity of mucosa composition**		
Impaired barrier of the small intestine due to increase of intestinal permeability and alteration of tight junctions.	CD	(15)
Inhibition of angiogenesis during ulcer healing by dysregulation of nitric oxide (NO) production.	Gastric ulcer	(10, 16)
Alteration of microvasculature, impairing vascular endothelial grown factor (VEGF) pathway, promotes ischemia in the gut.	Gastric ulcer and CD	(10, 15)
Cellular apoptosis due to increased reactive oxygen species and mechanical effects.	Gastric, esophageal and colon cancer	(17–19)
**Effects related with immune response**		
Induction of proinflammatory chemokines and cytokines (CCR6, CCL20, IL-8) in the ileum.	CD	(20)
Alteration of dendritic cell phenotype including an increase in the expression of MHC-II and costimulatory molecules.	UC	(21)
Increased recruitment of CD4^+^ and CD8^+^ T cells, and of CD11b^+^ dendritic cells on Ileum.	CD	(19, 22)

The intestinal effects produced by cigarette smoking could be partially related to the large amounts of particulate matter ingested by the smoker. It has been described that the amount of nicotine in gastric fluid is 10 times higher than in arterial blood and 80 times higher than in venous blood (23). Nevertheless, the gastrointestinal tract can also be affected by circulating components. Evidence suggests that chronic cigarette smoking (people who have smoked for more than 2 years) could increase the secretion of gastric acid and lower the stomach pH (5). In agreement with this idea, some studies have demonstrated a positive correlation between smoking and the probability of *Helicobacter pylori* infection, as well as with disease progression (24). In addition, chronic cigarette smoking seems to modify mucus production by the gastric (5) and intestinal mucosa (12), and to alter mucosal repair in the gut (5). Moreover, the vasoconstrictor and procoagulant characteristics of cigarette smoke can have different effects at the gastrointestinal level. For example, it has been described that chronic cigarette smoking alters microcirculation and significantly reduces blood flow to the gastrointestinal mucosa (25), which may favor the development of inflammatory diseases.

The role of cigarette smoking in intestinal inflammation has been extensively studied due to the contradictory effects observed in patients with IBD. IBD is a chronic disorder that usually begins in early adulthood, and whose symptoms include recurrent diarrhea, abdominal pain, and the presence of blood in stool (26). The two main disorders of IBD are CD (26) and UC (27). Although CD and UC share several characteristics, they differ in terms of clinical, endoscopic, and histological features (27). They also differ in their associated risk factors, such as cigarette smoking and some susceptibility loci. CD is characterized by an inflammatory process that can affect any portion of the gastrointestinal tract, from the mouth to the perianal area, in a discontinuous and transmural fashion. Depending on the location of the inflammation, CD has traditionally been classified into ileal, colonic, ileocolonic, and upper gastrointestinal phenotype (26). The most frequent phenotype among CD patients is the ileocolonic disease (26). Colonic inflammation manifests with more symptoms, whereas ileal inflammation seems to progress more rapidly toward transmural lesions, such as fistulas or stenosis (28).

Conversely, UC is a chronic and idiopathic inflammatory disorder of the colonic mucosa that begins in the rectum and usually extends proximally in a continuous manner through the entire colon or through a defined area. However, some patients with proctitis or left-sided colitis may have a cecal inflammation patch. Bloody diarrhea is the characteristic symptom of UC. The clinical course is unpredictable and is marked by alternating periods of exacerbation and remission (27).

In recent years, there has been a substantial improvement in the understanding of the pathophysiology of gastrointestinal inflammatory diseases; however, their etiological pathways remain unclear, and the incidence of CD and UC has increased markedly throughout the world. Unfortunately, there is still no cure for IBD (29). Several implicated environmental factors have been studied, cigarette smoking being the most widely described. Interestingly, although cigarette smoking increases the risk of CD, it is currently the epidemiological factor most associated with a lower incidence of UC (30). These contradictory effects are not fully understood; however, they seem to depend on etiological differences between both disorders, as well as on site-dependent impacts, which will be discussed in this review.

#### IBD: Dissimilarities between CD and UC

In recent years, three main factors have been identified that contribute to IBD pathogenesis: genetic factors, the host’s immune system, environmental factors, and gut microbiota (31). Some of the environmental factors that could affect the composition of intestinal microbiota and produce alterations in the immune system are changes in lifestyle, such as taking antibiotics, Western-style diet, and cigarette smoking. All of these could explain the increase in the incidence of CD and UC (32). It seems that in both disorders, genetically susceptible individuals have an inappropriate mucosal immune response against their gut microbiota, which leads to an aberrant inflammation response in the digestive tract (31). It has been shown that innate cells such as neutrophils, monocytes, macrophages, and dendritic cells, as well as non-immune cells such as epithelial and stromal cells, contribute to IBD pathogenesis by producing large amounts of cytokines (33). Moreover, it was found that mucosal dendritic cells express high levels of toll-like receptor (TLR) in both CD and UC, which can induce proinflammatory responses upon stimulation by enteric microbiota (33).

Crohn’s disease has been linked to more than 140 genetic susceptibility loci. About 30% of these loci are shared with UC, and about 50% of them are also shared with at least another immune-mediated disease (34). Moreover, predictive models based on genetic analysis can distinguish between colonic and ileal CD (35). Shared loci are enriched in genes involved in primary immunodeficiencies, T-cell function, and modulation of cytokine production (31). For instance, variants of IL23R and HLA are associated with both colonic CD and UC (31, 35). Remarkably, the strongest associations with extensive UC are with variants of the ancestral 8.1 HLA haplotype, which is a known recessive risk for primary sclerosing cholangitis, another inflammatory disease (35). On the other hand, CD seems to be more related to alterations in the immune response and effector functions involved in bacterial clearance. An example is the association between ileal CD and NOD2 variants (35). NOD2 encodes the primary receptor for muramyl dipeptide (MDP) and is essential for bacterial recognition. A loss of NOD2 function is a key event in the pathogenesis of ileal CD, since defective NOD2 leads to an increase in inflammation due to impaired bacterial clearance (36). In addition, a genome-wide association study identified an association between CD and variants of ATG16L1 and IRGM, two genes involved in autophagy (31). The susceptibility loci ATG16L1 directly affect the function of Paneth cells, which exhibit defects in the exocytosis of their granules (37) and affect, in the case of some specific mutations, their response to endoplasmic reticulum stress (38). The defects described above lead to a poor antimicrobial response, which allows the persistence of bacteria in the intestine lining (36, 38). This favors the activation of the immune response against the commensal microbiota. The identification of these genetic predispositions has allowed a deeper understanding of the aberrant response generated in CD and UC. However, genetic factors account for only about 20% of heritability in IBD (34).

The impaired immune response in susceptible individuals who develop IBD is essential to perpetuate intestinal inflammation. However, CD and UC have different adaptive immune responses. In CD, infiltrating CD4^+^ T cells produce a large number of Th1/Th17-associated pro-inflammatory cytokines such as IFN-γ, IL-17A, and TNF-α (26); and the secretion of these cytokines changes during the course of the disease. In the first stage, T cells secrete Th1 cytokines and TNF-α; in the transition stage, Th17 immune response begins with the secretion of IL-6 and IL-23; and finally, during the establishment of the disease, the combination of the Th1 and Th17 immune response is perpetuated, without TNF-α production (39). In UC patients, on the other hand, CD4^+^ T cells secrete Th2-associated proinflammatory cytokines, such as IL-4, IL-13, and IL-14 (27). IL-13 secretion promotes apoptosis of epithelial cells and alteration of their tight junctions (40, 41).

NKT cells are also an important part of the immune response in UC pathology: they secrete IL-13, which causes damage in the epithelium, and this consequently generates a positive feedback that increases NKT cell function (42). Interestingly, UC mucosa is specifically populated by type-II NKT cells that respond to a sulfatide autoantigen by producing IL-13. This suggests that this form of IBD is an autoimmune condition in which a self-glycolipid activates lamina propria NKT cells that mediate epithelial cell damage (43). However, one study shows that IL-13 is not related to UC immune response, because neutralization of this cytokine does not prevent or ameliorate the disease (44). More research is needed to solve this discrepancy.

It has been also suggested that humoral immunity has a more relevant etiological role in UC than in CD. Some studies have reported that intestinal damage and extra-intestinal manifestations present in patients with UC could be caused by an adaptive immune response mediated by antibodies that target autoantigens (45–47). Conversely, CD patients develop antibodies against bacterial antigens, suggesting a humoral response triggered primarily by impaired intestinal barrier function and, therefore, an increased interaction with the gut microbiota (48). In fact, the fundamental role of the disruption of the epithelium and the microbiota in the pathogenesis of CD has been widely described, both in human and animal models (48). Alterations in microbiota composition have been observed in both CD and UC. In CD, it has been reported an increase in Bacteroidetes, Proteobacteria, *Enterobacteriaceae, Pasteurellaceae, Vellonellaceae*, and *Fusobacteriaceae*, and a reduction in Firmicutes, Erysipelotrichales, and Clostridiales (33). In fact, patients suffering from ileal CD show a distinct change in their microbiota, characterized by a reduction of *Faecalibacterium* and *Roseburia* and an increase of *Enterobacteriaceae* and *Ruminococcus gnavus* (49). In UC, a change in the gut microbiota has also been reported, but less dramatic than that reported for CD. Here, sequence analysis of 16S rDNA revealed a reduction of Firmicutes and Bacteroidetes and an increase of Actinobacteria and Proteobacteria (33).

#### Opposite Effects of Cigarette Smoking on CD and UC

##### Epidemiology

Currently, it is widely accepted that cigarette smoking confers protection against UC (29). A meta-analysis confirmed that the risk of suffering UC is significantly lower in people who smoke, compared with people who have never smoked [OR 0.58 (0.45–0.75), (50)]. In addition, hospitalization and relapse rates, along with the need for more potent drugs and colectomy, are significantly lower in patients who smoke (30).

However, the protective effect of cigarette smoking in UC is temporary, since the relative risk of developing the disease increases after smoking cessation, compared with patients who have never smoked (OR 1.64 [1.36–1.98]) (51). In addition, cigarette smoking has been reported to increase the activity of the disease (52). Although the effects of smoking are not the same as those of nicotine, there is clinical evidence that suggests that nicotine and/or its metabolites, such as cotinine, are responsible for the beneficial effect of smoking in patients with active UC.

Nicotine has been used as a therapeutic agent in UC patients, and its administration has been in the form of chewing gum, transdermal patches, and nicotine-based enemas (53, 54). These studies indicated that chewing nicotine gum results in maximum levels of nicotine in the blood, similarly as those observed during smoking (55, 56). Moreover, a clinical trial with UC patients comparing the use of transdermal nicotine patches to placebo patches suggested that the direct use of nicotine is more effective in controlling mild-to-moderate colitis manifestations (57, 58). Although transdermal nicotine may be effective for UC, it has limited use due to its side effects such as acute pancreatitis, headache, and nausea (58). However, topical administration of nicotine into the colon by enema or administration of delayed-release oral capsule formulations decreases side effects, endoscopic characteristics, and histological damage in UC patients (58). Contradictorily, a recent study indicates that nicotine enemas do not provide benefits for the treatment of active UC; therefore, the benefits of these treatments are not yet conclusive (54).

On the other hand, early smoking significantly increases the risk of CD [OR 2.0 (1.65–2.47), (51)]. In addition, CD patients who smoke have a worse course of illness and quality of life, and are more likely to develop complications, have a higher rate of hospitalization, show a worse response to treatments, and have a greater need for surgery (59). Interestingly, there is a clear association between cigarette smoking and the site of inflammation in these patients, which mainly involves the ileum (ileal phenotype) (60). These associations have been confirmed in animal models (15). In addition, clinical and surgical recurrence rates are significantly higher in these patients, compared with non-smoking patients. In fact, 70% of CD patients who smoke and underwent ileal resection suffered macroscopic lesions at the site of anastomosis within the first year after surgery, as compared with only 35% of non-smoking patients (61).

As in UC, the impact of cigarette smoking on CD is temporary, and smoking cessation improves the course of the disease. In fact, it has been estimated that after 2 years of smoking cessation, the activity of the disease and the therapeutic requirement of former smokers are the same as those of patients who have never smoked (62, 63).

##### Proposed Mechanisms of Action

The pathways by which cigarette smoking exerts opposite effects in CD and UC are hard to establish, due to the large number of components in the smoke and the diverse cellular and tissue functions that may be affected, such as the mucosal barrier, gut microbiota, immune system, microvasculature, and others. Several studies have described the effect of cigarette smoking on the immune system. The first tissues that interact directly with cigarette smoke are the respiratory and gastrointestinal tract, where it can affect the local immune response. In agreement with this idea, it has been described that cigarette smoking is a risk factor for *Mycobacterium tuberculosis* (Mtb) infection and disease (64). One of the explanations for this susceptibility lies in an abnormal innate immune response. Smokers have significantly more alveolar macrophages (AM) than non-smokers and former smokers. In addition, AM from smokers cannot control intracellular Mtb and secrete less TNF-α, IFN-γ, and IL-1β after infection, which explains the susceptibility of smokers to Mtb infection and disease (65).

Neutrophils are involved in the protection and elimination of bacterial infections in lungs, due to their ability to form neutrophil extracellular traps (NETosis). In a recent study, human blood neutrophils were stimulated with phorbol ester (PMA for phorbol-12-myristate-13-acetate, a compound known for its ability to stimulate NETosis) in the presence or absence of cigarette smoke condensate (CSC). In the presence of CSC, neutrophils showed an inhibition of NETosis that correlated with an attenuation of oxygen consumption, suggesting a mechanistic relationship between these events. If operative *in vivo*, attenuation of NETosis due to cigarette smoke may impair host immune responses and increase the risk of respiratory tract infections (66). These impairments in the innate immune response can in turn cause an ineffective adaptive immune response.

In an Mtb infection model, continuous exposure to cigarette smoke locally impaired the accumulation of antigen-presenting cells (APC) and their production of TNF-α, IL-12, and RANTES (CCL5). It also impaired the recruitment of CD4^+^ IFN-γ^+^ T cells into the lung, and weakened the formation of granuloma. On the other hand, smoking cessation was found to help restore type-1 immunity by rapidly improving lung APC functionality, enhancing the recruitment of CD4^+^ IFN-γ^+^ T cells into the lung, and promoting the granuloma formation (67). In addition, the number of Treg cells and the expression of IL-10 and Foxp3 were lower in the lungs of mice exposed to cigarette smoke than in the control group. More important, the numbers of Tregs negatively correlated with the numbers of Th17 and Tc17 cells (68). These effects on the immune system fit nicely in the pathogenesis of CD, given that Th1/Th17 responses and impairment of bacterial clearance are essential factors for the development of inflammation.

**Figure 1:**
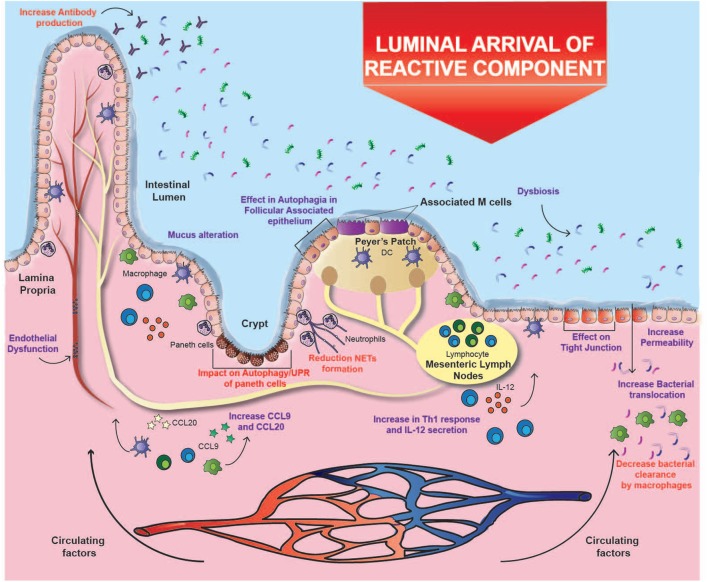
Effects of cigarette smoking on Crohn’s ileitis. A high percentage of particulate matter may reach the ileum, where it could alter the interaction of the intestinal mucosa with the microbiota through several mechanisms, e.g., affecting bacterial clearance, changing microbiota composition, increasing the permeability of the intestinal barrier, and disregulating immune responses. These alterations could prevail over the immunomodulatory effects of carbon monoxide and nicotine. Purple text denotes described effects and red text denotes proposed effects.

Cigarette smoke has an inhibitory effect on ulcer healing and repair of the gastrointestinal tract, possibly due to its toxic chemical components (10). It was found that the permeability of the intestinal barrier in healthy subjects is unaffected by smoking (69); however, an *in vitro* study with mice exposed to smoke described increased apoptosis in follicle-associated epithelium overlying Peyer’s patches. Moreover, total dendritic cells, macrophages, CD4^+^, and CD8^+^ T cells increased significantly after smoke exposure, together with an upregulation in mRNA expression of CCL9 and CCL20, two important chemokines in CD pathogenesis (19) (Figure 1). Additionally, one study demonstrated that cigarette smoking triggers colitis in mice mediated by CD4^+^ IFN-γ^+^ T cells (70). These results suggest that the injury caused in the epithelium barrier can lead to the exposure of lumen antigens, which can be recognized by immune cells whose recruitment is increased, inducing the development of CD (Figure 1).

As mentioned above, it is intriguing that despite the harmful effect of cigarette smoking in the gastrointestinal tract, it confers protection against UC. In this context, several inherent pharmacological properties of cigarette smoke could explain this. First, nicotine has an immunomodulatory effect mediated by the activation of nicotinic receptors α7 (α7-nAChR) in immune cells such as macrophages and dendritic cells. More specifically, stimulation of these receptors leads to decreased production of pro-inflammatory cytokines, both *in vitro* and *in vivo* (71). Additional evidence suggests that activation of α7-nAChR receptors is key in the immunosuppressive function of CD4^+^ CD25^+^ regulatory T cells, reducing NF-κB activation and IL-2 production (71). Other studies have shown that the α7-nAChR receptor is also expressed by endothelial cells, where its activation decreases the production of chemokines and the expression of adhesion molecules in the endothelium––a mechanism that could help to modulate leukocyte migration and inflammation (72). On the other hand, CO, the main component of the gas phase of cigarette smoke, has also been associated with anti-inflammatory properties. CO prevents the maturation of dendritic cells and reduces antigen presentation. In addition, it lowers the production of pro-inflammatory cytokines and the proliferation of effector T cells, whereas it stimulates the secretion of the anti-inflammatory cytokine IL-10 (73). Furthermore, exposure to CO causes improvements in genetic and chemical murine models of UC (73, 74). However, this anti-inflammatory effect predominates in UC but not in ileal CD, which could be explained by the different expression of receptors and other molecules by immune cells residing in the different parts of the intestine that are affected in CD or UC. An example of this is the different expression of aryl hydrocarbon receptor (AhR). Normal stimulation of this receptor by dioxin, a by-product of tobacco combustion, should induce anti-inflammatory and protective responses (75). However, it has been reported that the expression of AhR is downregulated in inflamed CD tissue compared with inflamed UC tissue (75).

In contrast to the beneficial effects observed in UC, the harmful effects of cigarette smoking prevail in ileal CD. Considering that CD development may be accompanied by multifocal microinfarcts in the gastrointestinal tract (76, 77), it seems logical that cigarette smoking can worsen the disease by increasing endothelial dysfunction. Accordingly, it has been described that the amount of oxidative and highly reactive compounds of cigarette smoke can intensify vasodilation malfunction in a chronically inflamed microvasculature; resulting in ischemia, ulcers, and fibrosis (4). Indeed, oxidative stress has been widely associated with CD, especially because enhanced oxidative stress and decreased antioxidant levels have been reported in patients with active CD (78). Interestingly, Li et al. demonstrated that cigarette smoke exposure increases oxidative stress in the small intestine of rats, causing upregulation of the nicotinamide adenine dinucleotide phosphate oxidase, while the antioxidative enzyme superoxide dismutase was downregulated (8). Again, this deleterious effect seems to predominate in CD, due to the impact it would have on bacterial clearance, as explained below.

The oxidative damage caused by cigarette smoke deteriorates lipids, proteins, DNA and even organelles; which finally undergo autophagy, a system of intracellular degradation that prevents cell death (19). In parallel to autophagy, intracellular damage can activate an interconnected response known as unfolded protein response (UPR), which is induced by reticulum stress (79). Interestingly, genes involved in both pathways (e.g., ATG16L1 and XBP1) have been associated with CD (80). Indeed, murine models with alterations in both pathways develop a phenotype that closely resembles the transmural inflammation observed in patients with CD (81). Therefore, the exhaustive use of these processes in response to damage caused by cigarette compounds could help explain the increased risk of developing CD, especially in genetically susceptible individuals (19). Remarkably, these interconnected pathways seem to be essential mechanisms in the immune response against pathogens (79). Consistently, Deuring et al. demonstrated that patients with an ATG16L1 risk allele present higher levels of reticulum stress markers and an altered intestinal microbiota (38). Therefore, cigarette smoke may alter the interaction between intestinal mucosa and microbiota through several mechanisms, including the effects mentioned above in autophagy and UPR (19) (Figure 1).

In addition, nicotine can affect bacterial clearance through the reduction of macrophages due to the activation of α7-nAChR (71). In fact, it has been shown that cigarette smoking significantly modifies the composition of intestinal microbiota by favoring the presence of opportunistic pathogens such as some species of *Enterobacteriaceae* and the phylum Bacteroidetes (13, 82). In relation to this, one study showed that the impact of smoking on the gut microbiota is temporary, since the amount of *Enterobacteriaceae* and Bacteroidetes decreases a few months after smoking cessation, while the abundance of some Firmicutes bacteria increases (13). Given that it is widely accepted that the disruption of the relationship between microbiota and intestinal mucosa is one of the main contributors to the pathophysiology of CD, the impact of cigarette smoking on the microbiota can have a great impact in the onset of this disease (Figure 1).

##### Etiological Differences or Site-Specific Impact?

**Figure 2:**
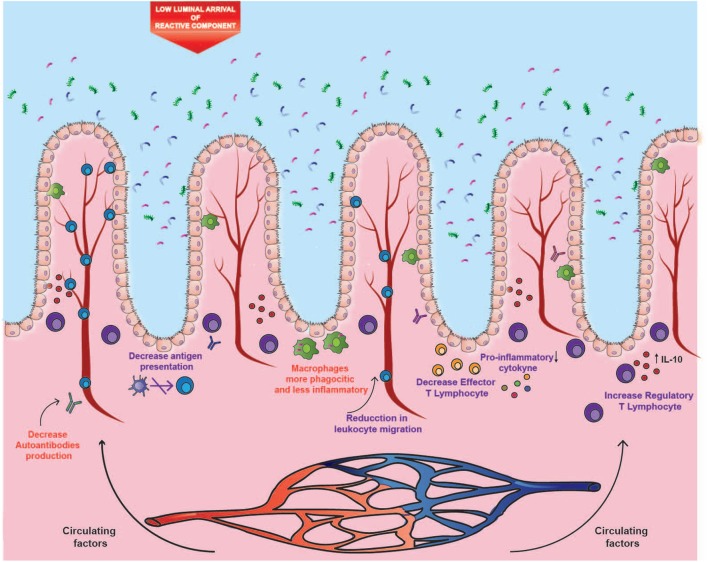
Impact of cigarette components on ulcerative colitis (UC). The distal segment of the large intestine could be mainly affected by the circulating components, where both carbon monoxide and nicotine could modulate the inflammatory profile, autoantibodies production, and leukocyte migration. These immunomodulatory effects could prevail in UC over the harmful effects observed in ileal Crohn’s disease, probably due to the lower arrival of luminal components and to the etiological differences between both disorders. Purple text denotes described effects and red text denotes proposed effects.

The opposite effects of cigarette smoking on ileal CD and UC could be explained by three different mechanisms. One of them is the etiological difference between both disorders. As mentioned above, the disruption of the intestinal mucosa and its interaction with a modified microbiota seems to be determinant in the etiology of CD. A murine study showed that exposure to cigarette smoke causes damage to the ileal mucosal barrier, generating greater permeability to bacteria (15) (Figure 1). Thus, it is likely that alterations of the gut microbiota caused by cigarette smoke have a more significant impact on CD. On the contrary, humoral immunity seems to play a more relevant role in the development and progression of UC (31, 46, 47). Therefore, in the intestinal mucosa, the reported immunomodulatory effects of nicotine and CO could prevail over the harmful impact of cigarette smoke in UC, but not in CD (Figure 2).

With respect to the location of inflammation, the opposite effects of cigarette smoke seem to be site-specific. In fact, murine studies have shown that exposure to cigarette smoke intensifies damage in the small intestine (15), whereas it reduces colitis (83). This site-specific effect could be given by a differential supply of cigarette compounds to the tissues, or by a tissue-dependent mechanism, thus originating the two remaining hypotheses. In this context, volatile compounds are rapidly absorbed through mucous membranes and alveoli, where they reach circulation. Conversely, a high percentage of particulate matter is swallowed, reaching high levels in saliva and gastric juices (23). In these fluids, several alkaline substances derived from tobacco accumulate because of their similar pH, and because they cannot be absorbed in their ionized form (23). Subsequently, these substances reach the small intestine, where they can be absorbed in the distal area as the pH increases, specifically in the ileum (84). As a second hypothesis, the considerable impact of cigarette smoke on the ileal mucosa can be attributed to the fact that this area suffers from a high exposure to these cigarette compounds (Figure 1). In fact, the distal segment of the large intestine can be affected mainly by the circulating components, where both CO and nicotine could modulate the inflammatory profile and leukocyte migration, and the immunomodulatory role would prevail over the impact on the mucosa (Figure 2).

Altogether, and as a third hypothesis, the harmful effects of cigarette smoking in the ileum could be tissue-dependent. In this context, some of the elements intrinsic to the ileum that could be affected by cigarette compounds are Peyer’s patches, Paneth cells, and tissue-specific microbiota (12, 19) (Figure 1). Interestingly, immune cells and Paneth cells present in Peyer’s patches are highly dependent on autophagy, and therefore could be severely affected by the reactive components of cigarette smoke (81). As a matter of fact, one study showed that mice exposed to cigarette smoke had a significant increase in autophagy levels in Peyer’s patches and in the surrounding epithelium (19), but there is still a lack of research on its actual effects on IBD patients. In fact, ileal epithelium and particularly Paneth cells are extremely sensitive to alterations in autophagy and UPR (81). As mentioned above, the impact of cigarette smoke on these pathways can lead to intestinal inflammation, impaired ileal barrier, and alterations in bacterial clearance (12, 19). Therefore, considering that there is a higher exposure to some cigarette components in that area, and that these processes are important in the pathophysiology of CD, the three hypotheses converge to help explain the increased risk of developing ileal CD in smokers.

## Conclusion

Although all the mechanisms mentioned above help explain the impact of cigarette smoke on CD and UC, they fail to explain the opposite effects on intestinal inflammation. In this scenario, there are three non-exclusive possibilities: the different etiology of both diseases, the location of the disease, and the differences in the immune response that are specific for the affected tissue. In addition, the interaction between genetic factors and other environmental aspects works in a complementary and non-exclusive way to promote the different effects of cigarette smoke on intestinal inflammation. Understanding the opposite effects of cigarette smoking on CD and UC will help to better understand the causes of these diseases, and the appropriate therapeutic approach to be used.

## Author Contributions

All authors listed have made a substantial, direct, and intellectual contribution to the work and approved it for publication.

## Conflict of Interest Statement

The authors declare that the research was conducted in the absence of any commercial or financial relationships that could be construed as a potential conflict of interest.
